# Assessing the contribution of alternative splicing to proteome diversity in *Arabidopsis thaliana *using proteomics data

**DOI:** 10.1186/1471-2229-11-82

**Published:** 2011-05-16

**Authors:** Edouard I Severing, Aalt DJ van Dijk, Roeland CHJ van Ham

**Affiliations:** 1 Applied Bioinformatics, Plant Research International, PO Box 619, 6700 AP Wageningen, The Netherlands; 2 Laboratory of Bioinformatics, Wageningen University, PO BOX 8128, 6700 ET Wageningen, The Netherlands; 3 Netherlands Bioinformatics Centre, PO BOX 9101, 6500 HB Nijmegen, The Netherlands; 4 Current address: Keygene N.V., P.O. Box 216, 6700 AE Wageningen, The Netherlands

## Abstract

**Background:**

Large-scale analyses of genomics and transcriptomics data have revealed that alternative splicing (AS) substantially increases the complexity of the transcriptome in higher eukaryotes. However, the extent to which this complexity is reflected at the level of the proteome remains unclear. On the basis of a lack of conservation of AS between species, we previously concluded that AS does not frequently serve as a mechanism that enables the production of multiple functional proteins from a single gene. Following this conclusion, we hypothesized that the extent to which AS events contribute to the proteome diversity in *Arabidopsis thaliana *would be lower than expected on the basis of transcriptomics data. Here, we test this hypothesis by analyzing two large-scale proteomics datasets from *Arabidopsis thaliana*.

**Results:**

A total of only 60 AS events could be confirmed using the proteomics data. However, for about 60% of the loci that, based on transcriptomics data, were predicted to produce multiple protein isoforms through AS, no isoform-specific peptides were found. We therefore performed *in silico *AS detection experiments to assess how well AS events were represented in the experimental datasets. The results of these *in silico *experiments indicated that the low number of confirmed AS events was the consequence of a limited sampling depth rather than *in vivo *under-representation of AS events in these datasets.

**Conclusion:**

Although the impact of AS on the functional properties of the proteome remains to be uncovered, the results of this study indicate that AS-induced diversity at the transcriptome level is also expressed at the proteome level.

## Background

Alternative splicing (AS) is a common phenomenon in higher eukaryotes that involves the production of multiple distinct mRNA molecules from a single gene. RNA-Seq surveys have shown that more than 90% of human and over 40% of *Arabidopsis thaliana *and rice genes are capable of producing multiple diverse mRNA molecules through AS [1-3]. A large fraction of AS events are predicted to result in transcripts that encode premature termination codons (see for instance [1,4]) and that are likely to be degraded through the nonsense mediated decay (NMD) pathway [5]. Although it has been the subject of several genome-wide studies (e.g. [6-8]), the extent to which the remaining fraction of AS events contribute to the functional protein repertoires of eukaryotes remains relatively unknown.

We concluded in a previous genome-wide comparative analysis of AS in three plant species that AS does not substantially contribute to functional diversity of the proteome [7]. Our conclusions were based on the limited conservation of AS events that can contribute to proteome diversity and the lack of conserved patterns that relate AS to gene function. Following this conclusion, it is conceivable that most AS events, in particular those that are not targeted towards NMD, result from noise in the splicing process [6] and are not strongly manifested at the protein level. However, lack of conservation can also mean that many protein isoforms have a confined, species-specific function rather than no function at all. In this scenario, it might be expected that most AS events are also expressed at the protein level. Determining which of these two scenarios is the most likely has been a difficult task because the majority of genome-wide studies of AS have been performed using protein isoforms deduced from transcriptomics data. For most of these isoforms no evidence for their expression at the protein level was available.

The gap between the availability of transcriptomics and proteomics data is steadily being bridged by the advancing field of mass spectrometry-based proteomics. This technology, which can be used to characterize complex protein mixtures [9], is of great value for studying the impact of AS at the proteome level. Indeed, a number of studies have appeared that describe the use of proteomics data for the identification of protein polymorphisms that are the result of AS [10-12].

In this study we address the impact of AS on proteome diversity in the model species *Arabidopsis thaliana *by reanalyzing the data from two independent large-scale proteomics studies [13,14]. Although AS was briefly addressed in these studies, their primary focus was on the confirmation and revision of existing gene structures and on the identification of new protein coding genes. The main objective of our study is to assess whether the predicted contribution of AS to the proteome diversity in *A. thaliana*, as based on transcriptomics data, is indeed observed at the proteome level.

We limited our study to those AS events that could be deduced from the annotated gene structures in the genome annotation database of *A. thaliana *version TAIR 10.0 (http://www.arabidopsis.org) and that are predicted to contribute to proteome diversity in this species. The absolute numbers of AS events that could be confirmed using the experimental peptide sets were by themselves not very indicative for the contribution of AS to the proteome diversity in *A. thaliana*. This is because these numbers depend on the depth of sampling in the experiments. We therefore performed *in silico *AS detection experiments using randomly generated peptide sets to assess the representativeness of the experimental sampling. This type of *in silico *experiments has previously been described and applied to *Drosophila *data [12].

We show that the outcome of the *in silico *experiments can lead to conflicting conclusions about the impact of AS on the proteome diversity, depending on the assumption that is used for generating the random peptide sets. We evaluate two of such assumptions and according to the biologically most realistic one, we show that AS events were not under-represented in the analyzed proteomics sets. This implies that variation due to splicing is to a large extent expressed at the proteome level.

## Results

Throughout this study we used three experimental datasets, the first two of which, hereafter referred to as the Castellana and Baerenfaller sets, contain peptides from two large-scale proteomics experiments on *A. thaliana *[13,14]. The third set, hereafter called the Merged set, was created by merging the Castellana and Baerenfaller sets into a non-redundant set. As it was essential for our study that each experimentally identified peptide could be reproduced by an *in silico *digestion of its parent protein, we only considered those peptides that met the following criteria: first, only one missed cleavage site (internal lysine or argine residues that were not used as cleavage sites by the trypsine enzyme) was allowed per peptide. Second, only those peptides that could be mapped to their parent proteins according to a strict set of rules were considered (see Material and Methods).

The initial set of annotated *A. thaliana *proteins (TAIR10.0) was also filtered by removing all proteins for which the exon/intron structure underlying its CDS region was not sufficiently supported by transcript data (see Material and Methods). The filtered protein set contained a total of 25,039 unique protein sequences derived from 21,136 nuclear-encoded, protein-coding TAIR 10.0 loci. Around 14.2% of the loci within the filtered protein set were predicted to produce distinct proteins through AS (hereafter called AS loci).

### Peptide mapping

The number of peptides that could be mapped back to TAIR 10 proteins (excluding chloroplast and mitochondrial encoded proteins) and the number of TAIR loci with at least one uniquely mapped peptide are summarized in Table 1. Although the number of mapped peptides from the Castellana set was slightly smaller than that of the Baerenfaller set, more loci were identified with the peptides from the Castellana set. However, the Castellana set was ~1.5 times larger than the initial Baerenfaller set (Table 1) and thus already represented more loci prior to the filtering step.

**Table 1:** Identification of nuclear encoded TAIR 10 loci.

Set	Total number of **peptides**^*a*^	Nr. of mapped peptides	% of peptides mapped	Nr. of TAIR loci identified	% of TAIR loci identified
Castellana	131,077	71,243	54.4	12,067	57.1
Baerenfaller	86,078	72,264	84.0	11,282	53.4
Merged	179,174	109,293	61.0	14,190	67.1

^***a ***^Totals refer to peptides containing at most one missed cleavage site

We note that a large fraction of the peptides from both the Baerenfaller (~16%) and Castellana (~45%) sets could not be mapped to any protein using our stringent criteria. These were kept stringent to ensure reproducibility of mapping results in the *in silico *experiments.

### AS detection results

**Figure 1:**
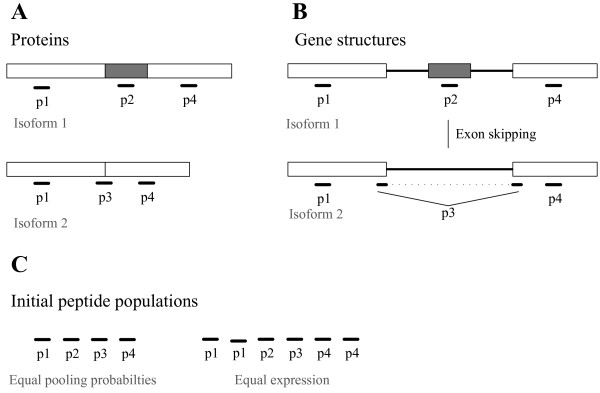
**Isoform and non-isoform specific peptides . (A) **Two protein isoforms (1 and 2) from an alternatively spliced gene that differ by a local polymorphism (inclusion/exclusion grey rectangle) yield two different peptide sets (Isoform 1: p1, p2, p4; Isoform 2: p1, p3, p4) when digested. While peptides p1 and p4 are non-specific because they map to both isoforms, peptides p2 and p3 are specific for isoform 1 and isoform 2, respectively. **(B) **The gene structures (exons correspond to the rectangles and the lines connecting them represent the introns) underlying these protein isoforms show that the AS event that is associated with the variable protein region is an exon-skipping event. In order to confirm the contribution of this specific exon-skipping event to the proteome diversity, both peptides p2 and p3 need to be identified. The dotted line indicates that p3 spans an exon/exon junction. (**C**) The initial peptide populations that are constructed for the *in silico *AS detection experiments differ under the two probability assumptions that are used in this study. Under the "equal pooling probability" assumptions, the initial peptide population consists of only unique peptides. Therefore the population contains only four different peptides. Under the "equal expression" assumption, the isoforms are represented by equal numbers of molecules prior to digestion. As a result, non-specific peptides are more abundant than isoform specific peptides.

AS events correspond to specific differences between the intron/exon architectures of two transcripts. If the AS event is located in the coding region of these transcripts, the resulting protein isoforms will in many cases differ by an indel (only these type of sequence variations were considered in this study). In order to confirm the contribution of a particular AS event to proteome diversity, peptides have to be identified that uniquely map to the variable protein regions that are associated with the AS event (Figure 1A and 1B). In addition, these peptides have to map according to a specific set of rules that differs per AS event type (Additional file 1, Figure S1). Due to the preference of trypsin to cleave after K- and R-residues [15], only a fixed number of peptides can, at least in theory, be obtained from a particular protein upon complete digestion. However, certain AS events may not be detectable because the peptides needed to confirm the events are not produced during digestion. Taken all together, the number of AS events that can be confirmed using proteomics data not only depends on the sampling depth and the number of co-expressed protein isoforms in a given sample, but also on the sequences of these proteins. For each of the experimental sets it was therefore determined what number of AS events could theoretically be confirmed (identifiable AS events). This was done by first performing an *in silico *digestion of all TAIR 10.0 proteins encoded by the loci that were expressed (represented by proteins) in the biological samples. The resulting *in silico *generated peptides were then mapped to their parent proteins and subsequently used for confirming AS events in the same way as was done for the experimentally identified peptides (Table 2).

**Table 2:** Experimentally confirmed AS events.

Set	AS loci	Identifiable AS events	AS loci w. confirmed AS events	(%)	Number of confirmed AS events	(%)
Castellana	1,434	1,789	38	2.6	38	2.1
Baerenfaller	1,318	1,641	21	1.6	21	1.3
Merged	1,644	2,059	59	3,6	60	2.9

For each experimental set, the number of TAIR 10 loci with identifiable AS events (AS loci) are given together with the number of identifiable AS events. Both the number of identifiable AS events that were confirmed using experimentally identified peptides and the number of AS loci with at least one confirmed AS events are provided. The percentages are fractions of AS loci and identifiable AS events.

A total of 38 AS events, corresponding to 38 AS loci were confirmed using the experimentally identified peptides from the Castellana set. Usage of the peptides from the Baerenfaller set resulted in the confirmation of 21 AS events from 21 AS loci (Table 2). Although more peptides from the Baerenfaller set could be mapped to their parent proteins than from the Castellana set, more AS events were confirmed using the latter set (Table 2). Comparison of the AS loci revealed that seven AS loci had confirmed AS events in both the Castellana- and Baerenfaller sets. In total, 60 AS events corresponding to 59 AS loci were confirmed using the experimental peptide set. These AS events represent ~2.9% of all AS events that could theoretically be confirmed using the merged peptide set. We note that for the Merged set the number of confirmed AS events was higher than the number of AS loci with confirmed AS events. This was due to a single AS locus that had more than one confirmed AS event. An overview of the annotations corresponding to the AS loci with confirmed AS events is provided in Additional file 2, Table S1.

### Sampling of AS regions

Next, we analyzed how well protein regions that corresponded to the location of AS events were sampled in each of the experimental sets. Here, sampling refers to the identification of peptides that map to either one of the two protein variants that are associated with an AS event. This is illustrated by the example shown in Figure 1A and B, in which either peptide p2 or p3 is identified, but not necessarily both. The analysis revealed that around 29% to 36% of AS events corresponding to ~31-38% of AS loci were sampled (Table 3).

**Table 3:** Sampling of AS events.

Set	Nr. of sampled AS events	% of identifiable AS events	AS loci w. sampled events	% of AS loci
Castellana	525	29.3	446	31.1
Baerenfaller	537	32.7	452	34.3
Merged	748	36.3	626	38.1

The percentage of identifiable events that have been sampled (i.e. at least one peptide is present that covers the region where AS induces local variation) and the percentage of AS loci with at least one sampled AS event are provided. The percentages in this table are relative to the number of identifiable AS events and AS loci for the corresponding sets as provided in Table 2.

### In silico *AS detection experiments*

**Figure 2:**
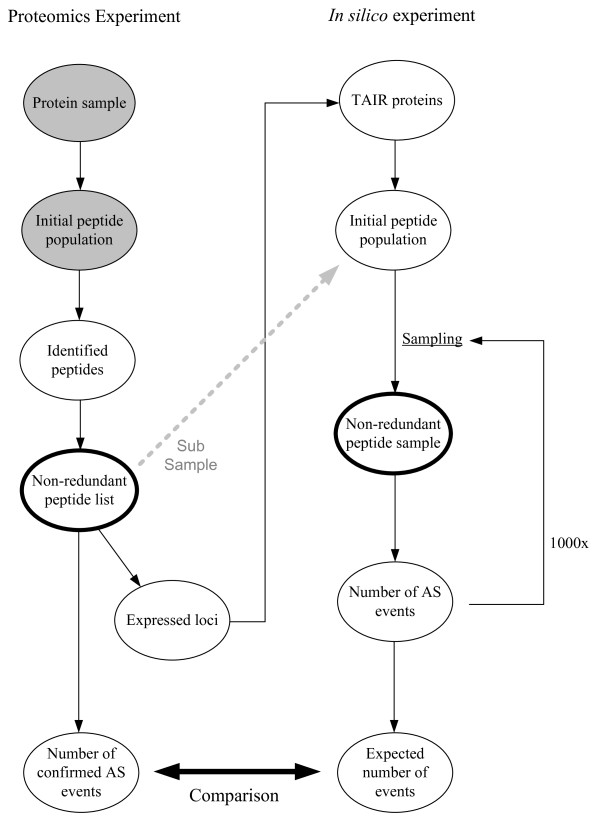
**Workflow for *in silico *AS detection experiments**. In the experimental proteomics study (left workflow), the (unknown) protein sample was digested using a protease enzyme. For a subset of the (unknown) initial peptide population the amino acid sequence was determined. This non-redundant peptide list was used for determining which loci were expressed (represented by a protein product) in the protein sample. The starting point for the simulations (right workflow) is a set of all annotated (TAIR) proteins encoded by the loci that were expressed in the biological sample. An initial peptide population was created by performing an *in silico *digestion of the set of annotated proteins. Note that the non redundant list of experimentally identified peptides is a subset of the *in silico *generated initial peptide population (grey dashed arrow). One thousand non-redundant peptide samples equal in size to the non-redundant list of experimentally identified peptides (thick lined ellipses in both workflows) were pooled from the initial peptide population. For each of the pooled peptide samples the number of AS events that could be confirmed with that sample was determined. Finally, the number of experimentally confirmed AS events was compared to the expected number of AS events which corresponds to the average number of AS events confirmed using the randomly generated peptide samples.

*In silico *AS detection experiments (Figure 2) were performed to assess how well AS events were represented in the experimental peptide sets. In brief, because of our strict mapping rules, all the experimental peptides that were considered in this study could be reproduced by performing an *in silico *digestion of the parent protein. As a result, each experimental peptide set was in fact a subset of an initial population that was generated by performing an *in silico *digestion of all annotated proteins encoded by the loci that were expressed in the biological samples. It was therefore possible for each of the experimental sets to test whether the number of confirmed AS events significantly differed from the number of events that can be expected to be confirmed using an equally sized, random subset of the same initial peptide population. The expected number of events corresponded to the average number of AS events that could be confirmed using 1000 randomly pooled peptide sets.

The composition of the random peptide sets and therefore also the AS detection outcome depends on the pooling probabilities that are assigned to the individual peptides in the initial *in silico *peptide populations. These pooling probabilities simply reflect the relative abundances of the peptides within the initial populations (see Material and Methods). We used two different assumptions for assigning pooling probabilities to the individual peptides (Figure 1C). The first assumption, to which we refer as the "*equal pooling probability*" assumption, has previously been described by Tress and co-workers [12]. Under this assumption, all peptides in the initial population are unique and therefore have the same probability of being pooled. Under the second assumption, hereafter referred to as the "*equal expression*" assumption, it was assumed that all genes were represented by equal numbers of protein molecules and that all isoforms of an AS locus were equally abundant in the protein sample. A consequence of this assumption was that the peptides within the initial populations were not equally abundant (Figure 1C).

Under the "*equal pooling probability*" assumption, the number of experimentally confirmed AS events in the Castellana set was 2.2 times smaller than the expected number of events as determined by the *in silico *experiments (Table 4; Simulations A). For the Baerenfaller and Merged sets, this same ratio was 4.8 and 2.7, respectively. Hence, when equal pooling probabilities are assumed, the *in silico *experiments indicate that AS events were under-represented in all experimental peptide sets.

**Table 4:** *In silico *AS detection experiments.

		Simulations A		Simulations B	
		
Set	Number of experimentally confirmed AS events	Mean nr. of AS events	SD	Mean nr. of AS events	SD
Castellana	38	85.4	9.3	20.5	4.7
Baerenfaller	21	100.4	10.1	26.0	5.2
Merged	60	160.6	12.9	39.7	6.4

The means and standard deviations are provided for the AS events that were confirmed in the in silico AS detection experiments. The experiments were performed under both the "equal pooling probability" (A) and "equal expression" (B) assumptions.

A different picture emerged from the simulations performed using the "*equal expression*" assumption. In this case, the number of experimentally confirmed AS events in the Castellana set was around 1.9 times larger than the expected number of events (Table 4; Simulations B). In contrast, the number of experimentally confirmed AS events for the Baerenfaller set fell within just 1 SD of the mean number of events as determined by the *in silico *experiments. Finally, the number of experimentally confirmed events for the Merged set was one and a half times larger than the expected number of events. In summary, under the "*equal expression*" assumption the *in silico *experiments indicate that; (*i*) AS events were not under-represented in the Baerenfaller set, and; (*ii*) AS events were over-represented in both the Castellana- and the Merged set.

### Disordered regions

**Figure 3:**
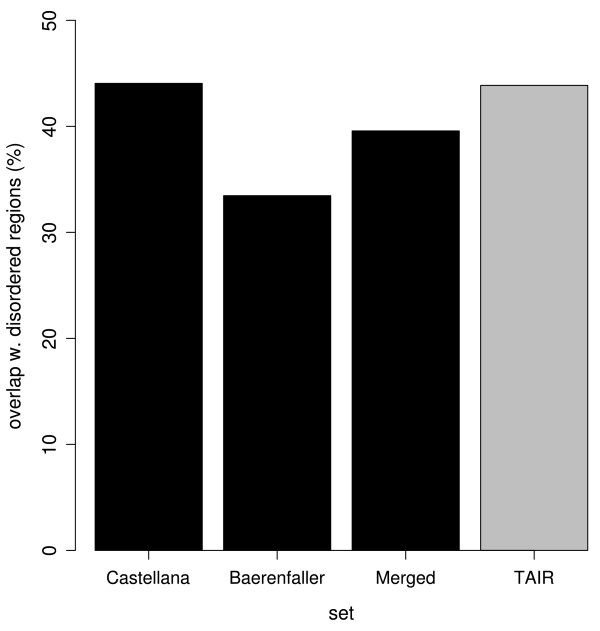
**Fraction of peptides that overlap with predicted disordered regions**. The fraction of peptides that overlap with disordered regions for all experimental sets (black) are shown together with the fraction of peptides generated through an *in silico *digestion of all nuclear encoded TAIR proteins that overlap with disordered regions (grey).

The peptides in both the Castellana and Baerenfaller set were extracted from different organs and cell cultures. However, the Castellana set also contained peptides that were derived from a phosphopeptide-enriched sample. It has previously been shown that phosphopeptide enrichment can result in an enhanced detection of AS events that are typically located within disordered regions of proteins [12]. Analysis of the protein regions to which the peptides from each experimental set were mapped indeed revealed a higher fraction of peptides mapping to disordered regions in the Castellana set than in the Baerenfaller set (Figure 3). For the Merged set this fraction fell, as expected, in between those for the Baerenfaller and Castellana sets. Comparison to the same fraction calculated for the TAIR set (peptides generated from all nuclear encoded TAIR proteins and mapping to disordered regions) revealed not much difference with the Castellana. However, the fractions for the Baerenfaller and Merged sets were smaller than the fraction for the TAIR set. Hence, compared to the TAIR - and Castellana sets, disordered regions were under-represented in the Bearenfaller and Merged sets.

**Figure 4:**
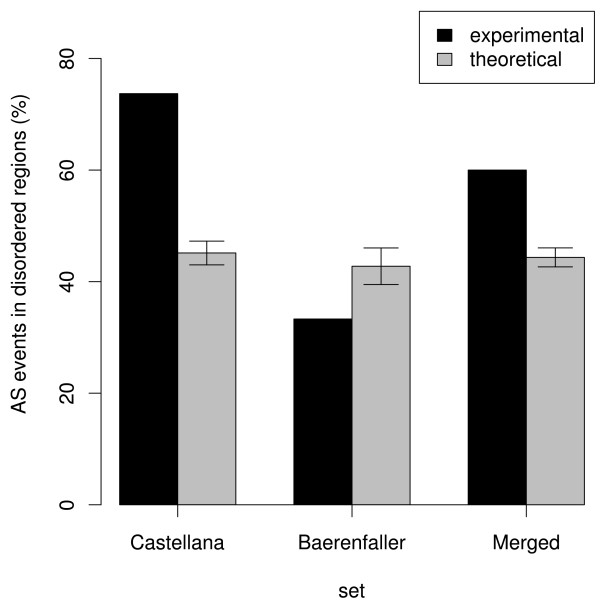
**AS events overlapping with disordered regions**. For all sets, the fraction of experimentally confirmed AS events that overlap with disordered regions (black) is shown next to the mean fraction of simulated events that overlap with disordered regions (grey). Error bars correspond to 1 SD from the mean.

Next, it was investigated whether the experimentally confirmed AS events were biased towards or against disordered regions, relative to expectation. To this end, the fraction of experimentally confirmed AS events from disordered regions was compared to a theoretical fraction. This theoretical fraction corresponded to the average fraction of AS events from 1000 randomly generated AS event sets (containing the same number of events as the corresponding experimental set) that overlapped with disordered regions. The AS events within these randomly generated sets were pooled from all identifiable AS events. Note that the number of identifiable AS events differs per experimental set. The results indicated that experimentally confirmed AS events were biased towards disordered regions in the Castellana set (Figure 4). Removal of all peptides containing phosphorylated residues (8,128 peptides) from the Castellana set did not affect this result (data not shown). In contrast, the fraction of confirmed AS events from the Baerenfaller set that were located in disordered regions was lower than its theoretical fraction. Finally, the fraction of AS events from the Merged set that were located in disordered regions was, similar as for the Castellana set, higher than its theoretical fraction. In summary, while the AS events in the Bearenfaller set were biased against disordered regions, the opposite was true for the AS events in the Castellana and Merged sets.

## Discussion

Genome-wide studies that address the impact of AS on proteome diversity have thus far mainly been performed using indirect evidence from transcriptomics data. Data that can be used to directly assess this impact is increasingly being provided by high-throughput proteomics experiments. Here we studied the impact of AS on proteome diversity in the model species *Arabidopsis thaliana *by reanalyzing data from two previous, large-scale proteomics studies [13,14]. The main goal of our study was to determine whether the contribution of AS events to proteome diversity as predicted using transcriptomics data, is indeed observed at the proteome level.

The absolute numbers of AS events that could be confirmed using the experimentally identified peptides were not particularly high and only represented around 2 to 3% of identifiable AS events. Analysis of the representation of protein regions corresponding to the location of AS events that were sampled in the experiments showed that for roughly two thirds of AS loci no peptides were detected that could discriminate between the different protein isoforms. The absolute numbers of confirmed AS *per se *are therefore not very indicative for the extent to which AS contributes to proteome diversity in *A. thaliana*.

We performed *in silico *AS detection experiments to determine how well AS events were represented in the biological samples, given the sampling depth achieved in the proteomics experiments. The *in silico *experiments should thus reveal whether the number of AS events identified using the experimental peptide sets significantly deviated from the expected number of AS events. The latter was calculated using an equally-sized random subset of *in silico *peptides pooled from the an initial peptide population. This initial peptide population consisted of all peptides that theoretically could be obtained through digestion of the proteins (including isoforms resulting from AS) that were encoded by the loci expressed in the experimental samples.

One factor that critically influenced the outcome of these *in silico *experiments involved the pooling probabilities that were assigned to the individual peptides in the initial population. We performed the *in silico *experiments using two different pooling probability assumptions. The first, "*equal pooling probability*" assumption, indicated that AS events were under-represented in all experimental peptide sets. In a previous proteomics study performed on *Drosophila *data, the same "*equal pooling probability*" assumption was used for generating peptide samples and determining the number of expected AS events [12]. The results in our study are comparable to those obtained for the Brunner set in that study.

The results of the *in silico *experiments were very different for the "*equal expression*" assumption. In this case, AS events were found to be over-represented in the Castellana and Merged sets, while for the Baerenfaller set, the number of experimentally identified AS events fell within 1 SD of the expected number of events. The observation that AS events were not under-represented in the experimental samples corresponds to the results of a recent study in which many AS transcript isoforms were shown to be actively translated [16].

The inconsistency between the conclusions obtained under the two pooling probabilities assumptions is the result of the fact that isoform-specific peptides associated with AS events have higher pooling probabilities under the "*equal pooling probability" *assumption than under the "*equal expression*" assumption. Under the first assumption, isoform-specific peptides and non isoform-specific peptides are equally abundant. In contrast, under the "*equal expression*" assumption, non isoform-specific peptides are more abundant than isoform-specific peptides (Figure 1C). This difference results in different pooling probabilities, in which the "*equal pooling probability*" assumption provides an upper bound to the expected number of AS events. The "*equal expression*" assumption, however, does not provide a corresponding lower bound, because it does not consider the relative expression levels between two or more AS isoforms. Indeed, the effect of lowering of the expected number of events would only further increase if unequal expression of isoforms would be taken into account and would therefore strengthen the conclusion that AS events were not under-represented in the experimental peptide sets.

Although neither of the two pooling probability assumptions is truly realistic in a biological sense, the "*equal expression*" assumption arguably provides the better approximation. This follows from the fact that isoform-specific peptides are necessarily less abundant than non-isoform specific peptides. Using Figure 1 as illustration, this can be understood by considering the total amount of peptides produced from a single locus, whatever the relative expression level of the two underlying isoforms is: the amounts of the constitutive peptides p1 and p4 will be the same and will always equal the sum of p2+p3. Given this reasoning, the conclusion derived under the "*equal expression*" assumption, namely that AS is over-represented, or at least not under-represented in the experimental proteomics datasets, is the most plausible.

A key factor that might explain the over-representation of AS events in the Castellana set compared to the Baerenfaller set, involves the bias of AS events towards disordered regions of proteins in the former set. AS events located within disordered regions can introduce variations that have a limited impact on protein folding [17]. Because cells have evolved mechanisms that can recognize and remove incorrectly folded proteins [18], AS events that have a limited impact on the protein structure are more likely to be viable and manifested at the protein level. In fact, it has recently been shown that pairs of AS isoforms, for which evidence was available that they were expressed, differed by polymorphisms that were more often located within disordered regions than expected [19].

One property of disordered regions is that they allow proteins to bind with multiple partners with high specificity and low affinity [20]. AS within such regions are interesting because they might play an important role in regulating protein-protein interactions.

## Conclusions

We conclude that the low numbers of AS events that could be confirmed using the proteomics datasets for *A. thaliana *are the result of a relatively low depth of sampling in the proteomics experiments. *In silico *AS detection experiments, performed under the assumption of equal expression of isoforms, indicate that AS events were not under-represented in the experimental peptide sets. An important implication of this is that much or all of the AS variation in *A. thaliana *that is expressed at the transcriptome level and not degraded through the NMD pathway, is also manifested at the proteome level. The true extent, however, to which AS variants are functional remains to be uncovered. Given that AS variation is not well conserved in plants [7], genome-wide expression of AS variation at the proteome level could point to the possibility that many of the AS events are associated with protein isoforms that either have a species-specific function or that are stable enough to escape rapid protein turnover.

## Methods

### Initial data

Peptide sequences from the study performed by Baerenfaller and co-workers [13] were obtained by querying the Pride database [21] using the available BioMart interface. Peptide sequences from the study of Castellana and co-workers [14] were downloaded from the webpage of the authors (site referenced in their publication). An additional peptide set was constructed by merging the Baerenfaller and Castellana peptide sets into a non-redundant set. Because trypsin was used for digesting proteins in both proteomics studies, peptides containing internal lysine (K) or arginine (R) residues that were not immediately followed by a proline (P) residue, were considered to be the result of missed cleavage sites. All peptides that contained two or more missed cleavage sites were discarded.

The predicted proteome of *Arabidopsis thaliana *version TAIR 10 was downloaded from http://www.arabidopsis.org. The information within the "confidenceranking_exon"-file (ftp://ftp.arabidopsis.org/home/tair/Genes/

TAIR10_genome_release/confidenceranking_exon) was used for filtering the proteome using the following criteria: (*i*) a protein encoded by a multi exon gene was only kept if all splice junctions located within the corresponding CDS region were supported by transcript data (mRNA) data, and; (*ii*) a protein encoded by a single exon gene was kept if at least 80% of the gene was supported by transcript data.

### Mapping peptides against their parent proteins

Vmatch (http://www.vmatch.de/) was used for performing exact searches with the peptides against the filtered proteome of *A. thaliana*. All matches were subsequently filtered using the following criteria: (*i*) peptides that did not map to the C-terminus of their parent protein were required to have a K- or R- residue at their C-terminus; (*ii*) peptide matches were discarded if the corresponding region of the parent protein was not immediately preceded by a K- or R-residue, unless the peptide mapped to the N-terminus of the parent protein; (*iii*) peptide matches were discarded if the corresponding region of the parent protein was immediately followed by a P-residue. Finally, only those proteins were considered that had at least one mapped peptide which was unique for the locus from which the protein originated.

### Identification of AS events at the proteome level

AS events were deduced from the annotated gene structures using a previously described method [7]. The identification of AS events at the proteome level was only performed with peptides that were unique for one or more, but not all of the protein isoforms of a locus. A schematic overview of the rules that were used for the identification of AS events at the proteome level is provided in Additional file 1, Figure S1.

### In silico *generation of peptide fragments*

Peptides were generated by performing an *in silico *trypsin digestion involving cleavage after K- and R- residues that were not followed by a P-residue. Only one missed cleavage site was allowed per peptide. All peptides with a mass outside the observed mass-range of the experimentally identified peptides (~523-5,399 Da and ~725-4,962 Da for the Castellana set and Baerenfaller set, respectively) were discarded.

### In silico AS detection experiments

The *in silico *AS detection experiments involved randomly pooling non-redundant peptide samples, equal in size to the experimental peptide samples, from an initial peptide population. This initial population only contained peptides that mapped to the protein products encoded by the loci which were expressed in the experimental samples. The probability of pooling a particular peptide depends on its abundance within the initial peptide population. The *in silico *detection experiments were performed using either one of the following two assumptions on the abundance of individual peptides within the initial peptide populations.

Under the first assumption to which we refer as the "equal pooling probability" assumption, all *in silico *generated peptides are equally abundant and therefore have the same probability (*1/N*) of being pooled, which depends on the size of initial peptide population (*N*). This pooling strategy, which has previously been described in [12], reflects a biological scenario in which individual proteins within an experimental sample are present in such numbers that subsequent digestion of the sample results in a population of equally abundant peptides.

Under the second assumption, to which we refer as the "*equal expression*" assumption, two basic rules are applied: (*i*) all genes are represented by equal amounts of protein molecules, and; (*ii*) all protein isoforms from an AS locus are present in equal numbers. The abundance of each protein within the sample is therefore determined as follows: Let *M *be the number of protein isoforms produced by the alternatively spliced gene with the highest number of unique protein isoforms. In order for rule (*i*) to be fulfilled, each gene has to produce *M *protein molecules. The protein product from a constitutively spliced gene is therefore present *M *times within the entire protein sample. To fulfill rule (*ii*), the number of molecules that correspond to a particular protein isoform of an AS locus that produces *X *different protein isoforms equals *M /X*. As a consequence, each peptide originating from this specific protein isoform is also represented by *M/X *molecules in the total peptide mixture after digestion. When for simplicity each peptide within the final sample is considered to be unique (even when multiple exact sequence copies exists), its pooling probability equals its abundance divided by the total number of peptides within the initial peptide population.

### Prediction of disordered regions

Putative disordered regions were predicted using the FoldIndex method [22] which is based on an algorithm developed by Uversky and co-workers [23]. In brief, the method uses hydrophobicity and net charge of protein sequence segments in order to distinguish disordered from ordered regions. By sliding over the protein sequences using a window of 51 AA and a step size of 1, disordered regions were identified as regions of at least five consecutive amino acid residues located in the centre of a window with a negative FoldIndex value.

## Authors' contributions

EIS conceived the experiments, carried out the study and drafted the manuscript. ADJvD participated in the design of the study and in drafting the manuscript. RCHJvH conceived of the study, participated in its design and coordination and helped to draft the manuscript. All authors read and approved the final manuscript.

## Supplementary Material

Additional file 1 **Figure S1 Figure S1**: Schematic overview of the rules used for detecting different alternative splicing events. Click here for file

Additional file 2 **Table S1. Table S1**: Annotation of loci with detected AS variants. Click here for file
