# Supplementary material for: Assessing the contribution of alternative splicing to proteome diversity in Arabidopsis thaliana using proteomics data
Source: BMC Plant Biol. 2011 May 16;11:82. doi: 10.1186/1471-2229-11-82 (PMC3118179; doi:10.1186/1471-2229-11-82)
Supplement: Additional file 1 — Figure S1 Figure S1: Schematic overview of the rules used for detecting different alternative splicing events. [file 1471-2229-11-82-S1.DOC]

**Figure S1. Detection of protein polymorphisms resulting from AS events**.

**A.** Detection of alternative acceptor events: Peptides (grey) are required that span both overlapping introns that differ in their acceptor sites. (The same rule is applied for alternative donor and position events). **B.** For the confirmation of exon skipping events it is required that at least one peptide (grey) is mapped to the skipped exon and one peptide spans the intron on the second isoform in which the skipped exon is fully engulfed. **C.** Intron retention events are confirmed when at least one peptide (grey) spans the retained intron and another peptide spans the spliced intron.
