# Supplementary material for: Assessing the contribution of alternative splicing to proteome diversity in Arabidopsis thaliana using proteomics data
Source: BMC Plant Biol. 2011 May 16;11:82. doi: 10.1186/1471-2229-11-82 (PMC3118179; doi:10.1186/1471-2229-11-82)
Supplement: Additional file 2 — Table S1. Table S1: Annotation of loci with detected AS variants. [file 1471-2229-11-82-S2.DOC]

**Table S1. Annotation of TAIR 10 loci with confirmed AS events.** For each locus is indicated whether AS events were confirmed in the Baerenfaller set (B), Castellana set (C), in both (Both), or in the Merged set (M).

| **TAIR locus** | **Peptide set** | **Description** |
| --- | --- | --- |
| AT1G08680 | C | Symbols: ZIGA4, AGD14 | ARF GAP-like zinc finger-containing protein ZIGA4 | chr1:2762820-2768387 FORWARD LENGTH=649 |
| AT1G14170 | C | Symbols: | RNA-binding KH domain-containing protein | chr1:4843463-4845253 REVERSE LENGTH=454 |
| AT1G17720 | Both | Symbols: ATB BETA | Protein phosphatase 2A, regulatory subunit PR55 | chr1:6093949-6098065 REVERSE LENGTH=501 |
| AT1G28530 | M | Symbols: | unknown protein; INVOLVED IN: biological_process unknown; LOCATED IN: chloroplast; EXPRESSED IN: 19 plant structures; EXPRESSED DURING: 10 growth stages; Has 20 Blast hits to 20 proteins in 6 species: Archae - 0; Bacteria - 0; Metazoa - 0; Fungi - 0; Plants - 20; Viruses - 0; Other Eukaryotes - 0 (source: NCBI BLink). | chr1:10032127-10035496 REVERSE LENGTH=614 |
| AT1G29040 | M | Symbols: | unknown protein; LOCATED IN: chloroplast, vacuole; EXPRESSED IN: 22 plant structures; EXPRESSED DURING: 13 growth stages; CONTAINS InterPro DOMAIN/s: Conserved hypothetical protein CHP02058 (InterPro:IPR011719); Has 344 Blast hits to 344 proteins in 122 species: Archae - 2; Bacteria - 227; Metazoa - 0; Fungi - 0; Plants - 32; Viruses - 0; Other Eukaryotes - 83 (source: NCBI BLink). | chr1:10134120-10135180 FORWARD LENGTH=198 |
| AT1G51690 | Both | Symbols: ATB ALPHA, B ALPHA | protein phosphatase 2A 55 kDa regulatory subunit B alpha isoform | chr1:19166218-19169974 FORWARD LENGTH=513 |
| AT1G58025 | C | Symbols: | DNA-binding bromodomain-containing protein | chr1:21458219-21461757 REVERSE LENGTH=572 |
| AT1G67500 | M | Symbols: ATREV3, REV3 | recovery protein 3 | chr1:25287707-25296714 REVERSE LENGTH=1890 |
| AT1G68010 | C | Symbols: HPR, ATHPR1 | hydroxypyruvate reductase | chr1:25493418-25495720 FORWARD LENGTH=386 |
| AT1G72640 | C | Symbols: | NAD(P)-binding Rossmann-fold superfamily protein | chr1:27346409-27348147 REVERSE LENGTH=312 |
| AT1G80810 | C | Symbols: | Tudor/PWWP/MBT superfamily protein | chr1:30365575-30368898 FORWARD LENGTH=773 |
| AT2G02390 | B | Symbols: ATGSTZ1, GST18, GSTZ1 | glutathione S-transferase zeta 1 | chr2:629015-630955 FORWARD LENGTH=221 |
| AT2G03640 | C | Symbols: | Nuclear transport factor 2 (NTF2) family protein with RNA binding (RRM-RBD-RNP motifs) domain | chr2:1104525-1106418 REVERSE LENGTH=422 |
| AT2G05520 | M | Symbols: GRP-3, ATGRP-3, GRP3, ATGRP3 | glycine-rich protein 3 | chr2:2026217-2026882 FORWARD LENGTH=145 |
| AT2G15490 | C | Symbols: UGT73B4 | UDP-glycosyltransferase 73B4 | chr2:6761750-6763398 FORWARD LENGTH=484 |
| AT2G16430 | C | Symbols: PAP10, ATPAP10 | purple acid phosphatase 10 | chr2:7120502-7122020 REVERSE LENGTH=348 |
| AT2G16600 | C | Symbols: ROC3 | rotamase CYP 3 | chr2:7200862-7201383 FORWARD LENGTH=173 |
| AT2G22620 | M | Symbols: | Rhamnogalacturonate lyase family protein | chr2:9604902-9610156 REVERSE LENGTH=677 |
| AT2G37340 | B | Symbols: RSZ33, ATRSZ33 | arginine/serine-rich zinc knuckle-containing protein 33 | chr2:15670372-15672331 REVERSE LENGTH=290 |
| AT2G39805 | C | Symbols: | Integral membrane Yip1 family protein | chr2:16610216-16612353 REVERSE LENGTH=275 |
| AT2G42590 | C | Symbols: GRF9, GF14 MU | general regulatory factor 9 | chr2:17732118-17733775 REVERSE LENGTH=263 |
| AT3G01500 | B | Symbols: CA1, ATBCA1, SABP3, ATSABP3 | carbonic anhydrase 1 | chr3:194853-196716 REVERSE LENGTH=270 |
| AT3G05420 | Both | Symbols: ACBP4 | acyl-CoA binding protein 4 | chr3:1561880-1567047 FORWARD LENGTH=668 |
| AT3G05760 | C | Symbols: | C2H2 and C2HC zinc fingers superfamily protein | chr3:1707981-1709910 FORWARD LENGTH=202 |
| AT3G06400 | C | Symbols: CHR11 | chromatin-remodeling protein 11 | chr3:1941066-1946700 FORWARD LENGTH=1055 |
| AT3G08770 | C | Symbols: LTP6 | lipid transfer protein 6 | chr3:2664349-2664784 REVERSE LENGTH=113 |
| AT3G10920 | B | Symbols: MSD1, MEE33, ATMSD1 | manganese superoxide dismutase 1 | chr3:3418015-3419581 FORWARD LENGTH=231 |
| AT3G12020 | M | Symbols: | P-loop containing nucleoside triphosphate hydrolases superfamily protein | chr3:3827016-3834146 FORWARD LENGTH=965 |
| AT3G13920 | B | Symbols: EIF4A1, RH4, TIF4A1 | eukaryotic translation initiation factor 4A1 | chr3:4592635-4594128 REVERSE LENGTH=412 |
| AT3G18860 | C | Symbols: | transducin family protein / WD-40 repeat family protein | chr3:6501774-6508352 FORWARD LENGTH=760 |
| AT3G49010 | C | Symbols: ATBBC1, BBC1, RSU2 | breast basic conserved 1 | chr3:18166971-18168047 REVERSE LENGTH=206 |
| AT3G51880 | Both | Symbols: HMGB1, NFD1 | high mobility group B1 | chr3:19247241-19248491 REVERSE LENGTH=178 |
| AT3G54440 | B | Symbols: | glycoside hydrolase family 2 protein | chr3:20148494-20157019 REVERSE LENGTH=1107 |
| AT3G57050 | B | Symbols: CBL | cystathionine beta-lyase | chr3:21111939-21114521 REVERSE LENGTH=464 |
| AT3G60240 | Both | Symbols: EIF4G, CUM2 | eukaryotic translation initiation factor 4G | chr3:22261842-22268295 FORWARD LENGTH=1723 |
| AT3G63460 | B | Symbols: | transducin family protein / WD-40 repeat family protein | chr3:23431009-23437241 REVERSE LENGTH=1104 |
| AT4G04830 | C | Symbols: ATMSRB5, MSRB5 | methionine sulfoxide reductase B5 | chr4:2445902-2446872 FORWARD LENGTH=139 |
| AT4G10480 | C | Symbols: | Nascent polypeptide-associated complex (NAC), alpha subunit family protein | chr4:6478089-6479079 REVERSE LENGTH=212 |
| AT4G16160 | B | Symbols: ATOEP16-2, ATOEP16-S | Mitochondrial import inner membrane translocase subunit Tim17/Tim22/Tim23 family protein | chr4:9157545-9158738 FORWARD LENGTH=176 |
| AT4G22350 | C | Symbols: | Ubiquitin C-terminal hydrolases superfamily protein | chr4:11804073-11806939 REVERSE LENGTH=510 |
| AT4G28706 | Both | Symbols: | pfkB-like carbohydrate kinase family protein | chr4:14167805-14170619 FORWARD LENGTH=401 |
| AT4G28820 | C | Symbols: | HIT-type Zinc finger family protein | chr4:14230815-14232287 REVERSE LENGTH=173 |
| AT4G32330 | C | Symbols: | TPX2 (targeting protein for Xklp2) protein family | chr4:15609801-15611867 FORWARD LENGTH=437 |
| AT4G34430 | C | Symbols: CHB3, ATSWI3D | DNA-binding family protein | chr4:16461069-16464993 FORWARD LENGTH=985 |
| AT5G08080 | B | Symbols: SYP132, ATSYP132 | syntaxin of plants 132 | chr5:2588532-2591106 FORWARD LENGTH=304 |
| AT5G09660 | B | Symbols: PMDH2 | peroxisomal NAD-malate dehydrogenase 2 | chr5:2993645-2995551 REVERSE LENGTH=354 |
| AT5G10470 | B | Symbols: KCA1, KAC1 | kinesin like protein for actin based chloroplast movement 1 | chr5:3290121-3297248 REVERSE LENGTH=1273 |
| AT5G13130 | C | Symbols: | Histidine kinase-, DNA gyrase B-, and HSP90-like ATPase family protein | chr5:4166755-4170324 FORWARD LENGTH=708 |
| AT5G20920 | C | Symbols: EIF2 BETA, EMB1401 | eukaryotic translation initiation factor 2 beta subunit | chr5:7094994-7096661 REVERSE LENGTH=268 |
| AT5G21060 | M | Symbols: | Glyceraldehyde-3-phosphate dehydrogenase-like family protein | chr5:7149153-7152745 REVERSE LENGTH=376 |
| AT5G27830 | C | Symbols: | FUNCTIONS IN: molecular_function unknown; INVOLVED IN: response to oxidative stress; LOCATED IN: endomembrane system; EXPRESSED IN: 22 plant structures; EXPRESSED DURING: 13 growth stages; CONTAINS InterPro DOMAIN/s: Folate receptor, conserved region (InterPro:IPR018143); Has 1807 Blast hits to 1807 proteins in 277 species: Archae - 0; Bacteria - 0; Metazoa - 736; Fungi - 347; Plants - 385; Viruses - 0; Other Eukaryotes - 339 (source: NCBI BLink). | chr5:9861344-9862742 FORWARD LENGTH=300 |
| AT5G38480 | C | Symbols: GRF3, RCI1 | general regulatory factor 3 | chr5:15410277-15411285 FORWARD LENGTH=255 |
| AT5G48230 | B | Symbols: EMB1276, ACAT2 | acetoacetyl-CoA thiolase 2 | chr5:19552570-19555030 REVERSE LENGTH=398 |
| AT5G55230 | C | Symbols: ATMAP65-1, MAP65-1 | microtubule-associated proteins 65-1 | chr5:22402716-22405182 FORWARD LENGTH=587 |
| AT5G58220 | Both | Symbols: TTL | transthyretin-like protein | chr5:23554546-23555861 REVERSE LENGTH=324 |
| AT5G61020 | C | Symbols: ECT3 | evolutionarily conserved C-terminal region 3 | chr5:24557485-24559780 REVERSE LENGTH=495 |
| AT5G61150 | C | Symbols: VIP4 | leo1-like family protein | chr5:24603846-24607528 REVERSE LENGTH=625 |
| AT5G64400 | B | Symbols: | CONTAINS InterPro DOMAIN/s: CHCH (InterPro:IPR010625); BEST Arabidopsis thaliana protein match is: Cox19-like CHCH family protein (TAIR:AT5G09570.1); Has 1807 Blast hits to 1807 proteins in 277 species: Archae - 0; Bacteria - 0; Metazoa - 736; Fungi - 347; Plants - 385; Viruses - 0; Other Eukaryotes - 339 (source: NCBI BLink). | chr5:25748922-25750182 FORWARD LENGTH=144 |
| AT5G66170 | C | Symbols: STR18 | sulfurtransferase 18 | chr5:26447828-26448582 FORWARD LENGTH=136 |
